# Plasma from Cardiac Surgery Patients Induces Endothelial and Tubular Epithelial Cell Damage: Potential Role in Acute Kidney Injury Development—A Preliminary Report

**DOI:** 10.3390/ijms27104416

**Published:** 2026-05-15

**Authors:** Elena Grossini, Teresa Esposito, Sakthipriyan Venkatesan, Mohammad Mostafa Ola Pour, Vincenzo Cantaluppi, Stefania Bruno, Daniela Ferrante, Veronica Daffara, Giulia Rizzotti, Daniele Pierelli, Jonathan Cattani, Carmelo Dominici, Antonio Nenna, Giovanni Casali, Gianmaria Cammarota, Rosanna Vaschetto

**Affiliations:** 1 Laboratory of Physiology, Department of Translational Medicine, Università Del Piemonte Orientale, Via Solaroli 17, 28100 Novara, Italy; sakthipriyan.venkatesan@uniupo.it (S.V.); 20046522@studenti.uniupo.it (M.M.O.P.); 2 Cardiac Anesthesia and Intensive Care Unit, Azienda Ospedaliero Universitaria Maggiore Della Carità, Corso Mazzini 18, 28100 Novara, Italy; expoterry@gmail.com (T.E.); veronica.daffara@maggioreosp.novara.it (V.D.); giulia.rizzotti@maggioreosp.novara.it (G.R.); daniele.pierelli@maggioreosp.novara.it (D.P.); 3 Nephrology and Kidney Transplantation Unit, Department of Translational Medicine, Azienda Ospedaliero Universitaria Maggiore Della Carità, Corso Mazzini 18, 28100 Novara, Italy; vincenzo.cantaluppi@med.uniupo.it; 4 Nephrology and Kidney Transplantation Unit, Department of Translational Medicine, Università Del Piemonte Orientale, Via Solaroli 17, 28100 Novara, Italy; 5 Department of Medical Sciences, University of Torino, 10127 Torino, Italy; stefania.bruno@unito.it; 6 Statistic Unit, Department of Translational Medicine, Università Del Piemonte Orientale, Via Solaroli 17, 28100 Novara, Italy; daniela.ferrante@med.uniupo.it; 7 Anesthesia and Intensive Care Unit, Azienda Ospedaliero Universitaria Maggiore Della Carità, Corso Mazzini 18, 28100 Novara, Italy; jonathan.cattani@maggioreosp.novara.it (J.C.); rosanna.vaschetto@med.uniupo.it (R.V.); 8 Cardiac Surgery Unit, Azienda Ospedaliero Universitaria Maggiore Della Carità, Corso Mazzini 18, 28100 Novara, Italy; cadominici@gmail.com (C.D.); antonio.nnn@hotmail.it (A.N.); giovanni.casali@maggioreosp.novara.it (G.C.); 9 Anesthesia and Intensive Care, Department of Translational Medicine, Università Del Piemonte Orientale, Via Solaroli 17, 28100 Novara, Italy; gianmaria.cammarota@uniupo.it; 10 Azienda Ospedaliero-Universitaria Santi Antonio e Biagio e Cesare Arrigo, Department of Anesthesia and Intensive Care, Via Venezia 16, 15121 Alessandria, Italy

**Keywords:** biomarkers, cardiopulmonary bypass, inflammation, oxidative stress, renal dysfunction, surgery

## Abstract

Cardiac surgery-associated acute kidney injury (CSA-AKI) is a frequent and severe complication of open-heart surgery. Although oxidative/inflammatory mechanisms are known to contribute to its pathophysiology, the circulating factors involved are poorly understood. In this preliminary investigation, we evaluated the effects of plasma from patients undergoing cardiac surgery on endothelial and renal tubular cells at anesthesia induction (T0) and 48 h after surgery (T1). Plasma levels of thiobarbituric acid-reactive substances (TBARSs), glutathione (GSH), and nitric oxide (NO) were measured in parallel. At T0, patient plasma showed increased TBARSs and reduced GSH and NO levels, consistent with oxidative imbalance, and induced cellular injury. In both cell types, plasma exposure reduced cell viability and mitochondrial membrane potential, while it increased oxidant release. Endothelial cells also showed decreased NO production, whereas renal tubular displayed increased apoptotic markers and reduced anti-aging factors. At T1, these alterations were further aggravated only in patients who developed CSA-AKI, whose plasma caused more severe endothelial and tubular damage. These findings support the presence of circulating injurious factors in cardiac surgery patient plasma that may contribute to CSA-AKI pathogenesis and help identify patients at risk before irreversible kidney damage develops.

## 1. Introduction

Acute kidney injury (AKI) is a clinical syndrome with variable etiology, defined as a rapid reduction in renal function caused by pathological conditions affecting nephron structure and function [1]. AKI is a common complication in patients undergoing cardiac surgery with extracorporeal circulation, affecting up to 30% of them and requiring renal replacement therapy (RRT) in 1–5% of cases, with a significant impact on morbidity and mortality [2,3,4,5]. The development of post-cardiac surgery AKI (CSA-AKI) is associated with prolonged mechanical ventilation, longer stays in intensive care units (ICUs) and hospital, and an increased risk of chronic RRT, including dialysis and kidney transplantation, with major economic and social consequences [6,7,8,9,10,11].

The pathophysiology of CSA-AKI is complex and involves renal ischemia–reperfusion injury, increased inflammatory response, oxidative stress, and hemolysis occurring after aortic cross-clamping and the initiation of cardiopulmonary bypass (CPB) [12,13,14,15,16,17,18,19,20]. Damage of both vascular endothelial cells and tubular cells plays a significant role in CSA-AKI onset. Indeed, renal hypoperfusion, resulting from the non-pulsatile blood flow that characterizes CPB, leads to endothelial dysfunction, reduced nitric oxide (NO) release, and vasoconstriction [16,17]. Endothelial dysfunction may be further aggravated by intravascular hemolysis caused by the contact of blood with the artificial surfaces of the extracorporeal circuit, as well as by inflammation and altered shear stress [14,17]. Concomitantly, prolonged renal ischemia may induce acute necrosis, particularly in the proximal tubule. Cellular injury, apoptosis, and/or necrosis can trigger the release of damage-associated molecular patterns (DAMPs), which in turn promote the production of inflammatory cytokines and complement proteins [18]. Finally, at the end of CPB, the reperfusion phase may further amplify renal damage through the activation of inflammatory pathways and the release of reactive oxygen species (ROS) [13,19].

Because CSA-AKI pathophysiology is multifactorial, its identification and management remain challenging. As there is still no consensus definition for CSA-AKI, the “Kidney Disease: Improving Global Outcomes” (KDIGO) diagnostic criteria are generally applied to this form of AKI as well [2,14,21,22]. Another major challenge is early diagnosis of CSA-AKI, which still relies largely on markers with poor diagnostic and prognostic accuracy, such as urine output and increased serum creatinine. Several additional biomarkers have been investigated to improve CSA-AKI diagnosis, including cystatin C, tumor necrosis factor alpha (TNF-α), neutrophil gelatinase-associated lipocalin (NGAL), interleukin 6 (IL-6), interleukin 18 (IL-18), kidney injury molecule-1 (KIM-1), liver-type fatty acid binding protein (L-FABP), N-acetyl-β-D-glucosaminidase, alpha-1 microglobulin, glutathione transferase-β, tissue inhibitor of metalloproteinase 1 and 2 (TIMP-1 and 2), and insulin-like growth factor-binding protein 7 (IGFBP7). However, none of the above has proven sufficiently effective for accurate diagnosis and reliable risk stratification of CSA-AKI [23,24,25].

Against this backdrop, the primary goal of this preliminary investigation was to examine plasma redox balance in patients undergoing cardiac surgery, both before and after CPB, and to evaluate the effects of patient plasma on vascular endothelial cells and renal tubular cells. Our hypothesis was that still-unidentified plasma circulating factors contribute to CSA-AKI pathogenesis and may also represent potential diagnostic and prognostic biomarkers. Accordingly, we investigated the relationship among plasma measurement results, in vitro findings and CSA-AKI development.

## 2. Results

### 2.1. Study Population

A total of 20 patients undergoing elective cardiac surgery were included in the study, of whom 10 developed CSA-AKI, whereas 10 did not. Baseline demographic characteristics were comparable between groups. Median age was 72 years in both AKI and non-AKI groups, and sex distribution was similar, with 30% females in each group. Body mass index (BMI), New York Heart Association (NYHA) functional class, left ventricular ejection fraction, and Thakar score were also comparable. Likewise, the prevalence of major cardiovascular risk factors, including hypertension, diabetes, chronic respiratory disease, and history of coronary artery disease or heart failure, did not differ significantly between groups.

At baseline (T0), patients who subsequently developed AKI had worse renal function, with higher serum creatinine levels and significantly lower estimated glomerular filtration rate (eGFR) than non-AKI patients (median eGFR 56 vs. 81 mL/min/1.73 m^2^, *p* = 0.0336). Chronic kidney disease was also more frequent in the AKI group (60% vs. 0%, *p* = 0.0108). Other baseline laboratory values and physiological parameters, including hemoglobin levels, PaO_2_/FiO_2_ ratio, lactate concentration, and mean arterial pressure, were similar between groups.

Surgical characteristics, including type of surgery, cardiopulmonary bypass duration, and aortic cross-clamp time, were comparable between AKI and non-AKI patients. Postoperative mechanical ventilation time and the incidence of perioperative complications also did not differ significantly. However, patients who developed AKI had longer ICU stays compared with non-AKI patients (median of 4 vs. 2 days, *p* = 0.002). Among AKI patients, those requiring RRT subsequently experienced recovery of renal function during the hospital stay. Of note, one patient died during the ICU stay due to progressive multi-organ failure following intraoperative aortic rupture and massive bleeding.

The healthy control (HC) group consisted of five patients undergoing elective non-cardiac surgery, with a median age of 70 years (IQR: 68–72), including three females and two males, and without major comorbidities.

### 2.2. Plasma Thiobarbituric Acid-Reactive Substances (TBARSs), Glutathione (GSH), and Nitric Oxide (NO)

**Figure 1:**
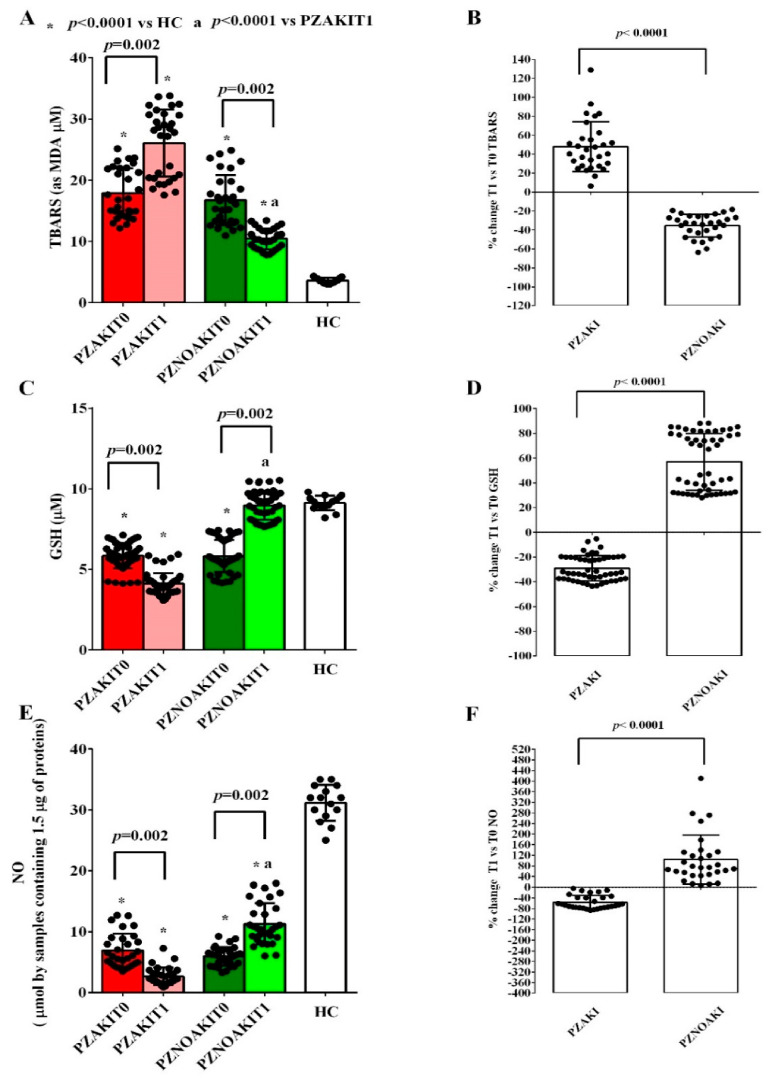
Plasma redox state in patients with or without AKI before and after cardiac surgery. Plasma levels of thiobarbituric acid-reactive substances (TBARSs), expressed as malondialdehyde (MDA), glutathione (GSH), and nitric oxide (NO) were measured in patients who developed acute kidney injury (AKI) and in patients who did not develop AKI (non-AKI) before cardiac surgery (T0) and 48 h after surgery (T1), as well as in healthy controls (HCs). Panels (**A**,**C,E**) show TBARS, GSH, and NO absolute values, respectively. Panels (**B**,**D**,**F**) show the percentage changes from T0 to T1 for TBARSs, GSH, and NO in AKI and non-AKI patients. The results are expressed as median and range of different measurements. The Wilcoxon signed-rank test and the Mann-Whitney test were used for the statistical analysis within the same group of patients and between the two groups of patients, respectively. A *p* value < 0.05 was considered for statistical significance. Square brackets indicate significant differences between groups (*p* < 0.05). Asterisks indicate significance vs. HC, whereas “a” indicates significance vs. PZAKIT1, as shown in the figure. PZAKIT0, plasma from AKI patients at T0; PZAKIT1, plasma from AKI patients at T1; PZNOAKIT0, plasma from non-AKI patients at T0; PZNOAKIT1, plasma from non-AKI patients at T1.

In order to analyze the redox state of patients at T0 (before surgery) and T1 (48 h after surgery), we quantified plasma TBARSs, GSH, and NO in the study cohorts and HCs. The results indicated that, even before surgery, patients displayed an altered redox state in comparison with that of HCs (Figure 1 and Figure S1). In particular, plasma GSH levels were lower, whereas TBARS levels were higher. Simultaneously, NO levels were reduced, suggesting endothelial dysfunction. No differences were observed at T0 between patients who subsequently developed AKI and those who did not. After 48 h from surgery (T1), redox imbalance and endothelial dysfunction were more pronounced in AKI patients, whereas an improvement was observed in non-AKI patients. Specifically, in AKI patients, plasma GSH and NO levels were lower, while TBARS levels were higher. The opposite was observed in non-AKI patients (Figure 1 and Figure S1).

### 2.3. Effects of Plasma on Human Umbilical Vein Endothelial Cells (HUVECs)

Because the plasma of cardiac surgery patients showed evidence of oxidative imbalance and reduced NO availability, we next investigated whether it could be associated with endothelial injury in vitro. In order to do this, we used two cell lines, HUVECs and renal tubular cells, which are representative of the glomerulus and the tubular system.

Although their use cannot reflect the complexity of the cardio-renal system in vivo, they still represent a validated investigation tool widely adopted to conduct studies related to renal function [26,27,28].

**Figure 2:**
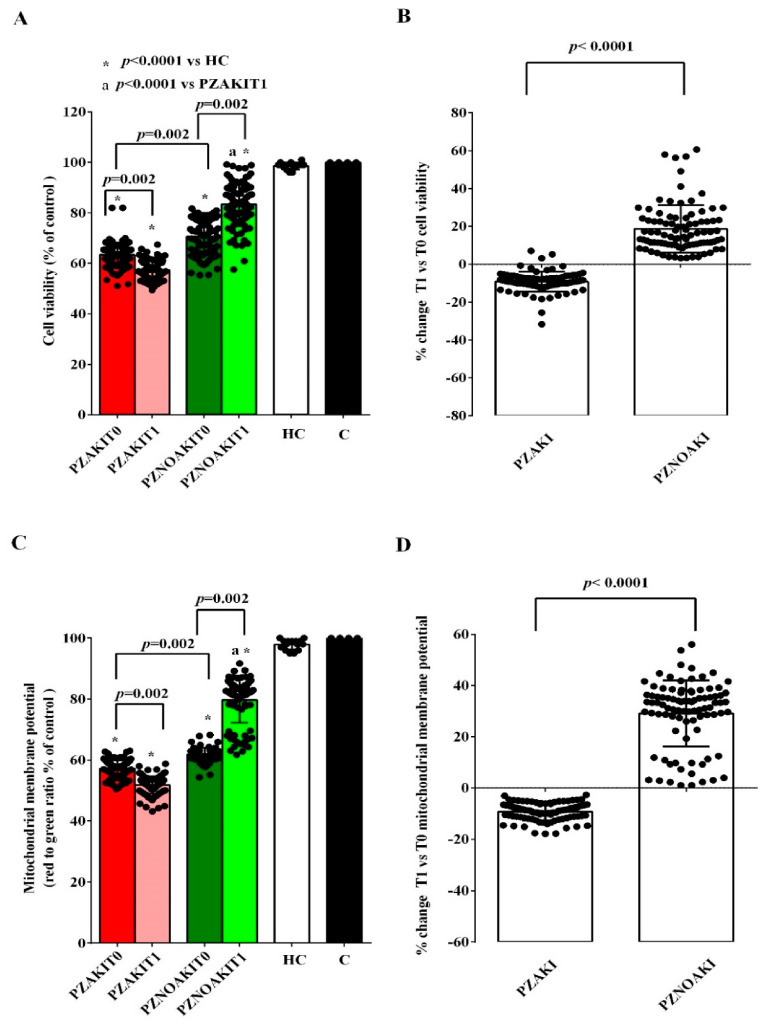
Effects of plasma from patients and HCs on cell viability and mitochondrial membrane potential in human umbilical vein endothelial cells (HUVECs). Cell viability (**A**,**B**) and mitochondrial membrane potential (**C**,**D**) were evaluated in HUVECs exposed to plasma from patients who developed acute kidney injury (AKI), patients who did not develop AKI (non-AKI), healthy controls (HC), and untreated cells (C). Panels (**A**,**C**) show absolute values in HUVECs treated with plasma collected before cardiac surgery (T0) and 48 h after cardiac surgery (T1). Panels (**B**,**D**) show the percentage changes between T0 and T1 in HUVECs treated with plasma from AKI and non-AKI patients. Results are expressed as median and range of different measurements. The Wilcoxon signed-rank test and the Mann–Whitney test were used for the statistical analysis within the same group of patients and between the two groups of patients, respectively. A *p* value < 0.05 was considered for statistical significance. Square brackets indicate significant differences between groups (*p* < 0.05). Asterisks indicate significance vs. HC, whereas “a” indicates significance vs. PZAKIT1, as shown in the figure. Other abbreviations: PZAKIT0, plasma from AKI patients at T0; PZAKIT1, plasma from AKI patients at T1; PZNOAKIT0, plasma from non-AKI patients at T0; PZNOAKIT1, plasma from non-AKI patients at T1.

**Figure 3:**
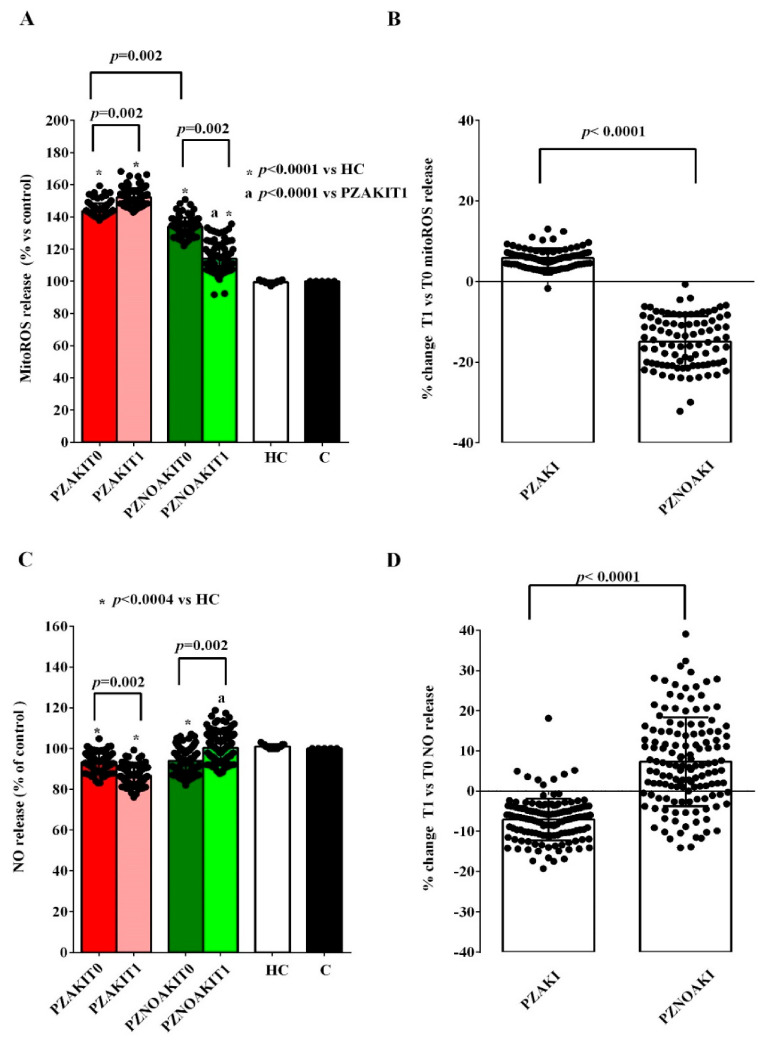
Effects of plasma from patients and HCs on mitoROS and NO release in human umbilical vein endothelial cells (HUVECs). Mitochondrial reactive oxygen species (mitoROS) release (**A**,**B**) and nitric oxide (NO) release (**C**,**D**) were evaluated in HUVECs exposed to plasma from patients who developed acute kidney injury (AKI), patients who did not develop AKI (non-AKI), healthy controls (HC), and untreated cells (C). Panels (**A**,**C**) show absolute values in HUVECs treated with plasma collected before cardiac surgery (T0) and 48 h after cardiac surgery (T1). Panels (**B**,**D**) show the percentage changes from T0 to T1 in HUVECs treated with plasma from AKI and non-AKI patients. Results are expressed as median and range of different measurements. The Wilcoxon signed-rank test and the Mann–Whitney test were used for the statistical analysis within the same group of patients and between the two groups of patients, respectively. A *p* value < 0.05 was considered for statistical significance. Square brackets indicate significant differences between groups (*p* < 0.05). Asterisks indicate significance vs. HC, whereas “a” indicates significance vs. PZAKIT1. Other abbreviations: PZAKIT0, plasma from AKI patients at T0; PZAKIT1, plasma from AKI patients at T1; PZNOAKIT0, plasma from non-AKI patients at T0; PZNOAKIT1, plasma from non-AKI patients at T1.

To this end, HUVECs were exposed to plasma collected at T0 and T1 from AKI and non-AKI patients. Treatment with patient plasma already at T0 was associated with lower cell viability and mitochondrial membrane potential (Figure 2 and Figure S2), decreased NO release, and increased mitochondrial reactive oxygen species (mitoROS) production (Figure 3 and Figure S2). These effects on cell viability, mitochondrial function, and oxidant release were more pronounced in HUVECs treated with plasma from AKI patients than in cells treated with plasma from non-AKI patients (Figure 2 and Figure 3).

**Figure 4:**
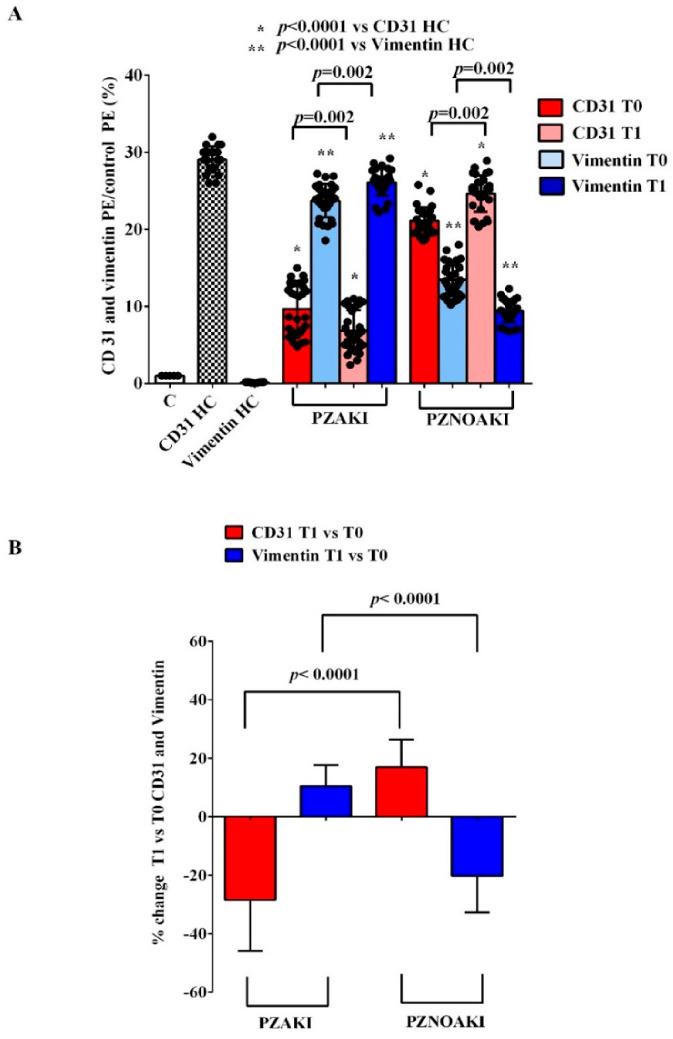
Effects of plasma from patients and HCs on EndMT markers in human umbilical vein endothelial cells (HUVECs). Endothelial-to-mesenchymal transition (EndMT) in HUVECs was evaluated by measuring the expression of CD31 and vimentin after exposure to plasma from patients who developed acute kidney injury (AKI), patients who did not develop AKI (non-AKI), and healthy controls (HC). (**A**) CD31 and vimentin expression in HUVECs treated with plasma collected before cardiac surgery (T0) and 48 h after cardiac surgery (T1) is shown. CD31 and vimentin phycoerythrin (PE) signals are expressed as the ratio vs. control PE. (**B**) percentage changes from T0 to T1 for CD31 and vimentin expression in HUVECs treated with plasma from AKI and non-AKI patients. Results are expressed as median and range of different measurements. The Wilcoxon signed-rank test and the Mann–Whitney test were used for the statistical analysis within the same group of patients and between the two groups of patients, respectively. A *p* value < 0.05 was considered for statistical significance. Square brackets indicate significant differences between groups (*p* < 0.05). Asterisks indicate significance vs. CD31 HC, whereas double asterisks indicate significance vs. vimentin HC. Other abbreviations: PZAKI, plasma from AKI patients; PZNOAKI, plasma from non-AKI patients.

At T1, the alterations observed in HUVECs exposed to plasma from AKI patients were more evident, as shown in Figure 4 and Figure S3. Indeed, cell viability, mitochondrial membrane potential and NO release were even more reduced, whereas mitoROS production increased compared with T0 (Figure 2 and Figure 3). By contrast, in non-AKI patients, all of these parameters showed an opposite trend, consistent with partial recovery (Figure 2 and Figure 3).

**Figure 5:**
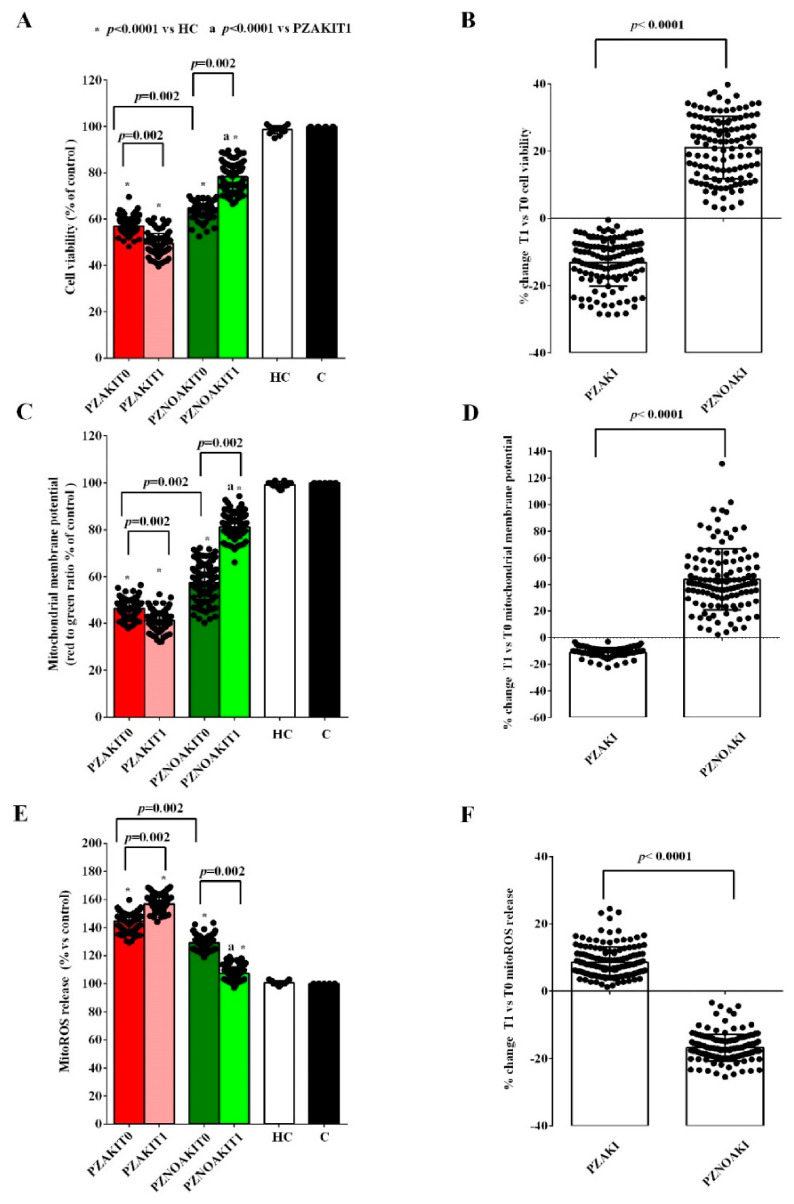
Effects of patient plasma on viability, mitochondrial membrane potential, and mitoROS release in renal tubular cells. Cell viability (**A**,**B**), mitochondrial membrane potential (**C**,**D**), and mitochondrial reactive oxygen species (mitoROS) release (**E**,**F**) were evaluated in renal tubular cells exposed to plasma from patients who developed acute kidney injury (AKI), patients who did not develop AKI (non-AKI), healthy controls (HC), and untreated cells (C). Panels (**A**,**C**,**E**) show absolute values in renal tubular cells treated with plasma collected before cardiac surgery (T0) and 48 h after cardiac surgery (T1). Panels (**B**,**D**,**F**) show the percentage changes from T0 to T1 in renal tubular cells treated with plasma from AKI and non-AKI patients. Results are expressed as median and range of different measurements. The Wilcoxon signed-rank test and the Mann–Whitney test were used for the statistical analysis within the same group of patients and between the two groups of patients, respectively. A *p* value < 0.05 was considered for statistical significance. Square brackets indicate significant differences between groups (*p* < 0.05). Asterisks indicate significance vs. HC, whereas “a” indicates significance vs. PZAKIT1. Other abbreviations: PZAKIT0, plasma from AKI patients at T0; PZAKIT1, plasma from AKI patients at T1; PZNOAKIT0, plasma from non-AKI patients at T0; PZNOAKIT1, plasma from non-AKI patients at T1.

The ability of patient plasma to influence endothelial damage was further confirmed by endothelial-to-mesenchymal transition (EndMT) analysis. As shown in Figure 4 and Figure S3, exposure of HUVECs to patient plasma resulted in lower expression of the endothelial marker CD31 and higher expression of the mesenchymal marker, vimentin, already at T0. At T1, the effects of plasma from AKI patients became even more evident. As shown in Figure 5, CD31 expression was further reduced, while vimentin expression was higher compared with findings at T0. In contrast, HUVECs treated with non-AKI patient plasma at T1 showed an improvement compared with T0. Specifically, CD31 expression was higher, whereas vimentin expression was lower.

### 2.4. Effects of Plasma on Renal Tubular Cells

We also analyzed the effects of plasma from patients and HCs on renal tubular cells. As reported in Figure 5 and Figure S4, plasma from patients showed lower cell viability and mitochondrial membrane potential, while displaying higher mitoROS. Consistent with the findings obtained in HUVECs, these effects were already more pronounced at T0 in renal tubular cells treated with plasma from AKI patients. At T1, the reduction in cell viability and mitochondrial membrane potential, as well as the increase in mitoROS release, became more evident in renal tubular cells treated with plasma from AKI patients (Figure 5). By contrast, an improvement was observed in renal tubular cells treated with plasma from non-AKI patients.

**Figure 6:**
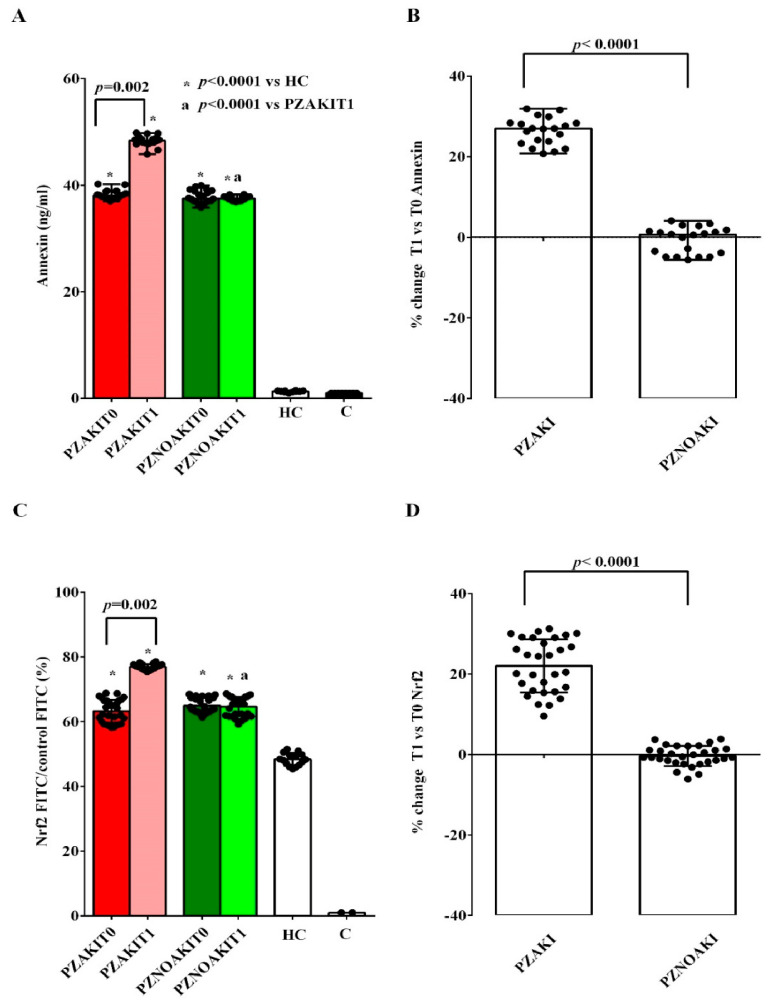
Effects of patient plasma on annexin and nuclear factor erythroid 2-related factor 2 (Nrf2) expression in renal tubular cells. Annexin expression (**A**,**B**) and Nrf2 expression (**C**,**D**) were evaluated in renal tubular cells exposed to plasma from patients who developed acute kidney injury (AKI), patients who did not develop AKI (non-AKI), healthy controls (HC), and untreated cells (C). Panels (**A**,**C**) show absolute values in renal tubular cells treated with plasma collected before cardiac surgery (T0) and 48 h after cardiac surgery (T1). Panels (**B**,**D**) show the percentage changes from T0 to T1 in fluorescein isothiocyanate (FITC) annexin and Nrf2 expression, respectively, in renal tubular cells treated with plasma from AKI and non-AKI patients. Results are expressed as median and range of different measurements; as regards Nrf2, the results are expressed as ratio vs. isotype control (control FITC) to set negative background levels. The Wilcoxon signed-rank test and the Mann–Whitney test were used for the statistical analysis within the same group of patients and between the two groups of patients, respectively. A *p* value < 0.05 was considered for statistical significance. Square brackets indicate significant differences between groups (*p* < 0.05). Asterisks indicate significance vs. HC, whereas “a” indicates significance vs. PZAKIT1. Other abbreviations: PZAKIT0, plasma from AKI patients at T0; PZAKIT1, plasma from AKI patients at T1; PZNOAKIT0, plasma from non-AKI patients at T0; PZNOAKIT1, plasma from non-AKI patients at T1.

The alterations observed in renal tubular cells were further evaluated by analyzing the apoptotic marker annexin, which showed higher expression in renal tubular cells treated with patient plasma than in those treated with HC plasma (Figure 6A,B and Figure S5). Notably, at T1, annexin expression was higher in renal tubular cells treated with plasma from AKI patients.

Interestingly, the expression of the antioxidant and pro-survival marker Nrf2 was already increased at T0 in the renal tubular cells treated with patient plasma and increased further at T1 in those exposed to plasma from AKI patients (Figure 6C,D and Figure S5).

**Figure 7:**
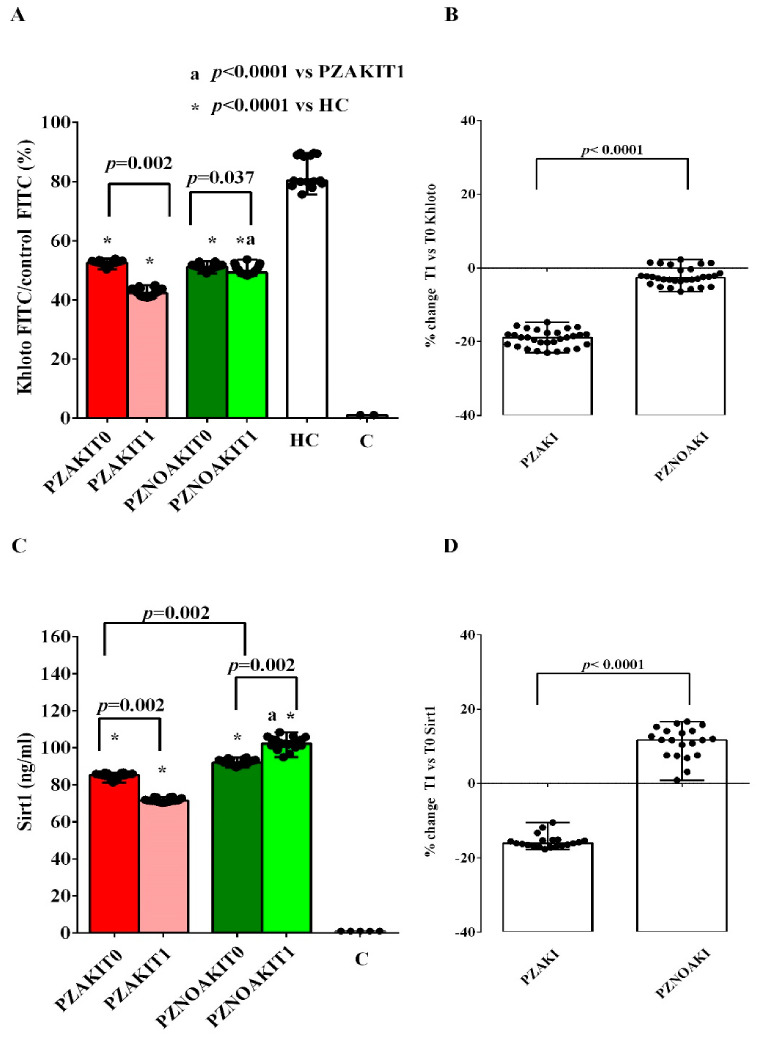
Effects of patient plasma on Klotho and sirtuin 1 (Sirt1) expression in renal tubular cells. Klotho expression (**A**,**B**) and Sirt1 expression (**C**,**D**) were evaluated in renal tubular cells exposed to plasma from patients who developed acute kidney injury (AKI), patients who did not develop AKI (non-AKI), healthy controls (HC), and untreated cells (C). Panels (**A**,**C**) show absolute values in renal tubular cells treated with plasma collected before cardiac surgery (T0) and 48 h after cardiac surgery (T1). Panels (**B**,**D**) show the percentage changes from T0 to T1 in fluorescein isothiocyanate (FITC) Klotho and Sirt1 expression, respectively, in renal tubular cells treated with plasma from AKI and non-AKI patients. Results are expressed as median and range of different measurements; as regards Khloto, the results are expressed as ratio vs. isotype control (control FITC) to set negative background levels. The Wilcoxon signed-rank test and the Mann–Whitney test were used for the statistical analysis within the same group of patients and between the two groups of patients, respectively. A *p* value < 0.05 was considered for statistical significance. Square brackets indicate significant differences between groups (*p* < 0.05). Asterisks indicate significance vs. HC in panel (**A**) and vs. C in panel (**C**), whereas “a” indicates significance vs. PZAKIT1. Other abbreviations: PZAKIT0, plasma from AKI patients at T0; PZAKIT1, plasma from AKI patients at T1; PZNOAKIT0, plasma from non-AKI patients at T0; PZNOAKIT1, plasma from non-AKI patients at T1; Sirt1, sirtuin 1; T0, before cardiac surgery; T1, 48 h after cardiac surgery.

Because cellular aging processes are known to be enhanced in AKI, we analyzed the expression of two main anti-aging factors, Sirt1 and Khloto, in renal tubular cells. As shown in Figure 7A,B and Figure S6, Klotho expression was lower in renal tubular cells treated with patient plasma than in cells treated with HC plasma. Moreover, Klotho expression was further reduced at T1 in cells treated with plasma from both AKI and non-AKI patients, although the reduction was greater in cells exposed to plasma from AKI patients (Figure 7A,B). Of note, the expression of the longevity marker Sirt1, was already lower at T0 in renal tubular cells treated with plasma from AKI patients than in cells treated with plasma from non-AKI patients and was further decreased at T1. By contrast, Sirt1 expression was higher in renal tubular cells treated with plasma from non-AKI patience (Figure 7C,D and Figure S6).

## 3. Discussion

The results of the present study show that plasma from patients undergoing cardiac surgery exerts cytotoxic effects on both HUVECs and renal tubular epithelial cells. These effects were already detectable preoperatively and became significantly more severe after surgery in patients who developed AKI. The harmful activity of patient plasma may be related to a pre-existing inflammatory/oxidative stress condition, as supported by the analysis of plasma redox state.

CSA-AKI is a rapid decline in kidney function occurring in the perioperative period and remains one of the most prevalent and severe complications of open-heart surgery [29]. It also represents the second most common cause of AKI in critically ill patients after sepsis [30]. As CSA-AKI is associated with high morbidity, high mortality, and substantial short- and long-term consequences [31], a better understanding of its pathophysiologic mechanisms would help support earlier diagnosis and more effective prevention. This need is reinforced by the limitations of current diagnostic tools, which still rely mainly on serum creatinine and urine output, two relatively insensitive and delayed markers [14]. In particular, serum creatinine rises only after substantial kidney injury has already occurred, and may be further masked by renal functional reserve (RFR). Other molecules, including NGAL, KIM-1, TIMP-2, IGFBP7, cystatin C, IL-6, and IL-18, have been extensively investigated as biomarkers of tubular epithelial cell stress and injury or as inflammatory indicators to identify high-risk patients and guide preventive interventions [23]. However, their performance has been inconsistent across studies and patient populations [32].

More specifically, the aforementioned limitations highlight the need to better define the early mechanisms that predispose patients to CSA-AKI and to clarify whether plasma alterations present before or shortly after surgery may contribute directly to renal injury. On this basis, we investigated whether patient plasma could induce direct endothelial and tubular injury in vitro. To this end, we examined the effects of plasma from patients undergoing cardiac surgery on HUVECs and renal tubular cells, two cellular components that are central to CSA-AKI pathogenesis.

Indeed, the endothelium plays a central and dynamic role in CSA-AKI pathophysiology, acting not only as a structural barrier but also as an active regulator of vascular tone, permeability, and inflammatory response. During cardiac surgery, the transition from physiological pulsatile flow to the laminar, non-pulsatile flow of CPB triggers an intense inflammatory response and reflex renal vasoconstriction [33]. This process leads to reduced NO release, which is essential for maintaining vascular homeostasis and adequate vasodilation. The loss of this vasodilatory function, combined with the shear stress generated by extracorporeal circulation and enhanced hemolysis, may severely compromise renal perfusion [34]. In addition, both ischemic injury and leukocyte activation increase vascular permeability and promote microthrombus formation, which can obstruct the microcirculation and further reduce oxygen delivery to renal cells [33,35].

Renal tubular cells, particularly those of the proximal tubule, are also primary targets of damage during and after cardiac surgery owing to their high metabolic activity and limited tolerance to oxygen deprivation [36]. During CPB, low-flow, non-pulsatile circulation may reduce oxygen supply and ATP availability, thereby promoting tubular necrosis and apoptosis. These effects are further intensified by endothelial dysfunction, which compromises renal microcirculation and worsens tissue hypoxia. During reperfusion after CPB, activated leukocyte infiltration and massive ROS generation may further aggravate injury in both endothelial and tubular cells, thus perpetuating a cycle of inflammation, oxidative stress, and cell death [34,35].

Our findings show that plasma from patients induces marked dysfunction in both endothelial and renal tubular cells, as indicated by reduced mitochondrial function and increased production of mitoROS [36]. In HUVECs, we also observed reduced NO release and activation of EndMT, as shown by reduced expression of the endothelial marker CD31 and increased expression of vimentin [37]. In renal tubular cells, patient plasma promoted apoptosis and reduced the expression of anti-aging factors such as Klotho and Sirt1, both of which are important for stress resistance and kidney homeostasis. As regards Klotho, studies showed it may have a role in reducing kidney damage and promoting kidney recovery. Similarly, SIRT1 has been reported to exert cytoprotective effects in the kidney by inhibiting cell apoptosis and inflammation [38,39,40]. Notably, Nrf2 expression increased in tubular cells, likely as an adaptive response to oxidative and inflammatory stress, consistent with its established antioxidant and pro-survival role [41].

An important aspect of this study is that many of these alterations were already evident before surgery and became more pronounced at 48 h after surgery in patients who developed AKI. The findings obtained at T0, therefore, support the existence of still-unidentified circulating plasma factors capable of inducing endothelial and tubular damage even before the surgical insult. These observations are consistent with our plasma measurements of redox states and NO levels. Indeed, already before surgery, patient plasma showed higher TBARS levels and lower GSH and NO levels than plasma from HCs. Overall, these data suggest that the altered balance between oxidant and antioxidant factors could be involved in damage to HUVECs and tubular cells because it is related to unclear circulating factors and still potentially capable of inducing those damages. It is well known that in aging and in pathological conditions such as ischemic heart failure, hypertension, renal failure and cardiac valvular disease [42,43,44], inflammation and oxidative stress can also play a central role through changes in the release of circulating factors, such as pro-inflammatory cytokines such as tumor necrosis factor α and interleukin 1 and 6, chemokines such as monocyte chemoattractant protein-1, and the complement system, which could contribute to systemic and intrarenal inflammation and oxidative stress. Also, vasoactive molecules and hormones, and damage-associated molecular patterns and toxins, could also induce oxidation and further renal damage [45,46,47]. Among possible factors, circulating extracellular vesicles (EVs) could play a role by potentially amplifying systemic and kidney damage and contributing to the progression of kidney disease, too [44]. Indeed, EVs could propagate inflammation and oxidative stress throughout the organism by delivering cargo enriched with pro-senescent and pro-inflammatory microRNAs and proteas part of the senescence-associated secretory phenotype. Moreover, mitochondrial DNA content within EVs can significantly decline with age and in pathologic conditions, adversely affecting mitochondrial energetics and cellular communication [48].

In this way, all above mentioned circulating factors could represent good candidates as mediators of renal damage and systemic evolution in CSA-AKI patients and their removal from plasma could represent a therapeutic tool for prevention and treatment of early AKI. However, although EVs and pro-inflammatory cytokines are compelling candidates, further studies are required to confirm their presence and activity within CSA-AKI patients.

The stronger damaging effects of patient plasma on endothelial and renal tubular cells observed at T1 may reflect quantitative and/or qualitative changes in the pool of circulating factors generated by the aforementioned pathophysiological mechanisms underlying CSA-AKI. A plausible explanation is that extracorporeal circulation and the postoperative phase, through renal hypoperfusion followed by blood flow restoration, promote mitochondrial structural damage, mitochondrial membrane potential loss, and enhanced oxidative stress. This aspect is particularly relevant because mitochondria and oxidative stress are tightly linked in CSA-AKI pathophysiology and sustain a vicious cycle of cellular damage affecting both renal tubular cells and the vascular endothelium [36,49]. However, this worsening was confined to patients predisposed to AKI. In the AKI group, plasma redox state deteriorated further at T1 and NO levels declined, whereas the opposite pattern was observed in non-AKI patients. These opposite trends in oxidant/antioxidant balance and NO levels support the hypothesis that changes in the pool of still-unidentified circulating factors associated with a pro-inflammatory and oxidative milieu may account for the greater plasma-induced cellular damage observed only in AKI patients at T1.

From a clinical perspective, the two study groups were largely comparable in terms of age, sex distribution, type of surgical procedure, CPB duration, aortic cross-clamp time, and major comorbidities traditionally associated with CSA-AKI, including diabetes, hypertension, chronic respiratory disease, and left ventricular dysfunction. Similarly, perioperative management, such as hemodynamic parameters and exposure to potentially nephrotoxic drugs, did not differ significantly between AKI and non-AKI patients.

Among the established risk factors for CSA-AKI, such as advanced age, female sex, obesity, prolonged CPB duration, intraoperative hypotension, anemia, and transfusion requirement, only pre-existing chronic kidney disease differed between the two groups. Patients who subsequently developed AKI already exhibited impaired renal function at baseline, as reflected by lower eGFR values and a higher prevalence of CKD. This finding is particularly relevant given that pre-existing CKD is recognized as the strongest and most consistent predictor of CSA-AKI [50]. In this context, RFR, defined as the difference between basal GFR and peak GFR after a stress test such as amino acid infusion, has proved highly predictive of AKI development in cardiac surgery patients. Of note, patients with preoperative RFR <15 mL/min/1.73 m showed an 11.8-time-increased risk of AKI during hospitalization [51]. Furthermore, among elective cardiac surgery patients, AKI development or even the presence of subclinical AKI—identified by positivity for tubular stress biomarkers—was associated with a significant reduction in RFR three months after surgery [52]. Taken together, these results suggest that the lower eGFR values detected before surgery in the AKI group may reflect reduced RFR and, consequently, decreased nephron mass.

Our data also suggest that baseline renal dysfunction may reflect a broader state of systemic vulnerability, characterized by chronic inflammation, oxidative stress, and endothelial dysfunction, which is already detectable at the plasma level before surgery. Importantly, although some patients in the AKI group required continuous RRT during the postoperative course, renal function recovery was observed in all cases during hospitalization, indicating a potentially reversible pattern of kidney injury.

Based on these observations, the improvement in redox state, NO levels, and plasma-induced cellular effects observed only in non-AKI patients at T1, despite otherwise comparable perioperative conditions, may be related to the different baseline renal function of the two groups. In patients who subsequently developed AKI, pre-existing renal dysfunction, inflammation, and oxidative stress may have acted as a priming condition that amplified the effects of cardiac surgery.

Taken together, these findings support a “two-hit” model of CSA-AKI. In patients with preserved renal function, cardiac surgery induces a transient inflammatory and oxidative insult that can be compensated and progressively resolved. By contrast, in patients with pre-existing renal impairment, a chronic pro-oxidative and pro-inflammatory milieu appears to be present already before surgery, as evidenced by altered plasma redox markers and cytotoxic effects on endothelial and tubular cells at T0. In this susceptible population, cardiac surgery may act as a second hit, amplifying mitochondrial dysfunction, oxidative stress, and cellular injury, ultimately leading to persistent AKI. This model is consistent with the divergence observed at 48 h postoperatively in plasma redox state and plasma-induced cellular effects between AKI and non-AKI patients.

Within this interpretive framework, a major strength of the present study is the integration of clinical data with mechanistic in vitro findings. Thanks to its multidisciplinary design, we were able to integrate clinical and laboratory expertise, thereby providing a broader view of CSA-AKI pathophysiology. In addition, the evidence that patient plasma exerts cytotoxic effects already before surgery addresses a critical clinical gap, because traditional markers increase only after substantial damage has already occurred. Although the specific circulating mediators were not identified, the demonstration of plasma-induced endothelial and tubular injury supports the existence of early pathogenic signals that may help identify patients at risk before kidney damage becomes irreversible. Finally, the combined use of HUVECs and renal tubular cells provides an experimental model that recapitulates the interaction between circulating plasma factors and two key cellular targets of kidney injury.

This study also has limitations that should be acknowledged. First, the relatively small sample size limits the generalizability of the results. However, the use of strict inclusion and exclusion criteria allowed the enrollment of a clinically homogeneous population, reducing heterogeneity and minimizing major confounding factors. In addition, the two patient groups were comparable for most established clinical and surgical risk factors for CSA-AKI, including age, sex, type of surgery, cardiopulmonary bypass duration and perioperative management, thereby strengthening the internal validity of the biological findings. Moreover, the results we obtained are the median of multiple replicates conducted on different cell lines and with small delta variations and for this reason we can be quite confident in the robustness of our data. Second, plasma samples were collected at two predefined time points (before surgery and 48 h after surgery). Although this design captured early postoperative changes associated with AKI development, it does not fully describe the temporal evolution of circulating cytotoxic factors. Earlier and serial sampling could better clarify the dynamics of plasma-mediated injury. Third, even though in vitro experiments using human endothelial and renal tubular cells provide mechanistic insights into plasma-induced cytotoxicity, these models cannot fully reproduce the complexity of the in vivo renal microenvironment, including immune interactions, neurohumoral regulation, and hemodynamic influences. They represent a validated tool for the analysis of kidney function in both physiologic and pathologic conditions [26,27,28]. Fourth, the study was not designed to identify the specific circulating mediators responsible for the observed effects. Plasma is a complex biological matrix, and the deleterious cellular responses likely result from the combined action of multiple inflammatory, oxidative, and possibly vesicle-associated factors, which warrant further investigation. Finally, although pre-existing renal dysfunction was associated with enhanced plasma cytotoxicity, causality cannot be established. Even so, these findings support the concept that baseline renal impairment reflects a state of systemic vulnerability characterized by chronic inflammation and oxidative stress, which may predispose patients to CSA-AKI following cardiac surgery. Moreover, although renal function recovered during hospitalization, the persistent reduction in anti-aging markers such as Klotho and Sirt1 in plasma-treated tubular cells indicates that molecular alterations may persist beyond clinical improvement. This sustained suppression of cytoprotective pathways suggests that subclinical cellular stress may continue after apparent renal recovery and could represent a sign of persistent subclinical damage and of poor reserve, with a greater risk for the onset of chronic disease. This aspect warrants further investigation, which could be performed in next studies with long-term follow-up.

## 4. Materials and Methods

### 4.1. Study Design

This study included consecutive patients undergoing elective cardiac surgery between 15 December 2024 and 30 March 2025 at the Cardiac Surgery and Cardiac Anesthesia and ICU of the Azienda Ospedaliero-Universitaria (AOU) Maggiore della Carità, Novara. The project was approved by the Ethics Committee of the AOU Maggiore della Carità (registration number: 845/CE). To ensure confidentiality, each participant was assigned a randomly generated alphanumeric code for sample collection and data analysis.

All patients met the inclusion criteria outlined below and provided written informed consent, either personally or through a legal representative when appropriate. This study was conducted in accordance with the principles of the Declaration of Helsinki (1964) and its subsequent amendments, or comparable ethical standards. For each patient, the following variables were collected: demographic characteristics, comorbidities, ongoing medications, admission diagnosis, routine laboratory and radiological findings, and AKI risk stratification using the Thakar Score or Cleveland Clinic Score [53].

Five patients undergoing elective non-cardiac surgery and without major comorbidities were enrolled as HCs, matched to the study population for age and sex.

Inclusion criteria were age ≥ 18 years and elective cardiac surgery, including coronary artery bypass grafting, valve replacement or repair, combined procedures, or ascending aortic surgery.

Exclusion criteria included emergency surgical procedures (e.g., aortic dissection or cardiac rupture), chronic dialysis treatment or advanced chronic kidney disease defined as KDIGO stage G4–G5 (stage G4: glomerular filtration rate [GFR] 15–29 mL/min/1.73 m^2^; stage G5: end-stage kidney disease with GFR < 15 mL/min/1.73 m^2^), active sepsis, and active infective endocarditis. In the operating room, before the start of the surgical procedure (T0), plasma samples (approximately 10 mL) were collected. The same sampling was repeated 48 h after the end of surgery (T1), during the postoperative stay in the Cardiac Surgery Intensive or Intermediate Care Unit of the AOU Maggiore della Carità Hospital, Novara. Plasma samples were used to assess TBARSs, GSH, and NO, as well as to perform in vitro experiments.

Throughout the perioperative period, all patients underwent continuous monitoring of vital parameters, ventilatory settings, and arterial blood gas analysis (ABG). Routine laboratory tests were performed according to standard ward protocols, including measurements of common AKI biomarkers: serum creatinine, electrolytes, lactate, calcium, C-reactive protein (CRP), procalcitonin, creatine kinase (CK), total and fractionated bilirubin, aspartate aminotransferase (AST), alanine aminotransferase (ALT), and troponin T (TnT). The development of AKI was defined according to the KDIGO criteria, based on changes in serum creatinine and/or urine output [22].

### 4.2. Collection of Blood Samples

Blood samples were collected from patients under fasting conditions using BD Vacutainer tubes containing sodium heparin as anticoagulant at T0 and T1. Samples were immediately centrifuged in a refrigerated centrifuge (Eppendorf, mod. 5702 with rotor A-4-38, Hamburg, Germany) at 3100 rpm for 10 min at 4 °C. The resulting plasma was aliquoted into five tubes and stored at −80 °C at the Physiology Laboratory of the Università del Piemonte Orientale. Plasma samples were further processed for the quantification of redox state markers, as specified below, and for in vitro experiments on HUVECs and renal tubular cells. Blood samples from HCs were collected and processed following the same procedure.

### 4.3. TBARS Quantification

Plasma TBARS levels were evaluated using the TBARS assay Kit (Cayman Chemical, Ann Arbor, MI, USA), which measures malondialdehyde (MDA) release [54,55,56]. For this assay, 100 μL of each plasma sample was added to sodium dodecyl sulfate solution (100 μL) and Color Reagent (2 mL). Each sample was boiled for 1 h and then placed on ice for 10 min to stop the reaction. Thereafter, each sample was subjected to centrifugation (10 min at 1600× *g*, 4 °C). After that, 150 μL was transferred to 96-well plates for MDA detection using a spectrophotometer (VICTOR™ X Multilabel Plate Reader, PerkinElmer; Waltham, MA, USA) at excitation/emission wavelengths of 530–540 nm. To quantify TBARS levels in each sample (expressed as μM of MDA), a standard curve was prepared using the TBARS Standard. Each measurement was performed in triplicate. Assay detection range: 0.625–50 μM.

### 4.4. Gsh Quantification

GSH measurement was performed in plasma using the Glutathione Assay Kit (Cayman Chemical, Ann Arbor, MI, USA) [54,55,56]. Briefly, each plasma sample was deproteinated by adding meta-phosphoric acid solution in an equal volume. After centrifugation at 2000× *g* for 2 min, the supernatant of each sample was collected and 50 μL/mL of Triethanolamine (TEAM) reagent was added to increase the pH. A total of 50 μL sample was transferred to 96-well plates where the measurement of GSH was carried out using a spectrophotometer (VICTOR™ X Multilabel Plate Reader, PerkinElmer; Waltham, MA, USA) with excitation/emission wavelengths of 405–414 nm. For accurate GSH quantification (μM), a standard curve was prepared using the GSH Standard. Each measurement was performed in quintuplicate. Assay detection range: 0.5–16 μM.

### 4.5. No Quantification

Plasma NO was quantified using the Griess assay (Promega Italia Srl, Milan, Italy) [54,55,56]. To achieve this, 5 mL plasma sample was deproteinated by adding 10 mL sulfosalicylic acid. The samples were then vortexed every 5 min and allowed to react for 30 min at room temperature. After centrifugation (10,000× *g* for 15 min), 50 μL of the supernatant was added to saline (1:2 dilution) for the subsequent analysis. The remaining supernatant was used without dilution. To reduce nitrate to nitrite, the samples were passed through a copper–cadmium column of an autoanalyzer (Autoanalyzer; Technicon Instruments Corp., Tarrytown, NY, USA) and then mixed with an equal volume of Griess reagents. After 10 min, the absorbance was measured using a spectrometer (VICTOR™ X Multilabel Plate Reader) at 570 nm. Each measurement was performed in triplicate. NO concentrations were quantified using a standard curve and expressed as nitrites (μM).

### 4.6. Effects of Plasma Samples on HUVECs and Renal Tubular Cells

**Figure 8:**
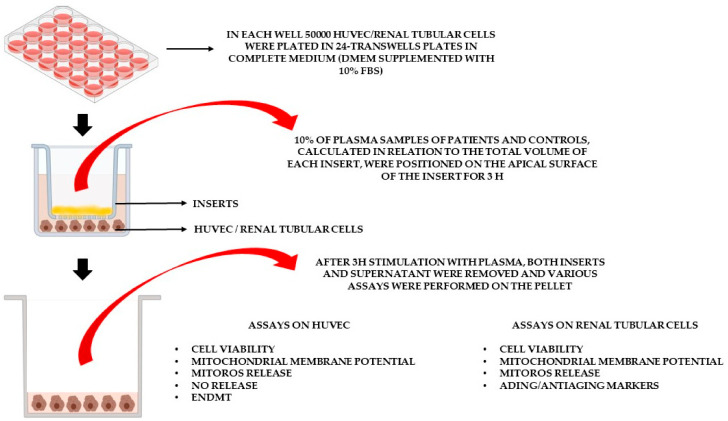
In vitro experimental protocol. DMEM: Dulbecco’s Modified Eagle Medium; EndMT: endothelial-to-mesenchymal transition; FBS: fetal bovine serum; HUVECs: human umbilical cord-derived endothelial vascular cells; MitoROS: mitochondrial reactive oxygen species; NO: nitric oxide.

For the in vitro experiments, plasma samples from 10 AKI patients at T0 and T1, 10 non-AKI patients at the same time points, and 5 HCs were used. Specific Transwell inserts were employed to evaluate the effects of plasma on cell viability, mitochondrial membrane potential, mitoROS production, NO release, and EndMT in HUVECs. In parallel, the effects of plasma were also examined on renal tubular cells as regards cell viability, mitochondrial membrane potential, and mitoROS release, as well as nuclear factor erythroid 2–related factor (Nrf2), Klotho, annexin V and sirtuin 1 (SIRT1) (Figure 8). As described in previous experiments [54,55,56], plasma samples (10% of the total volume of each insert) were placed in the apical chamber of the insert for 3 h, while HUVECs or renal tubular cells were plated in the basal one. After 3 h stimulation with plasma, the inserts were removed and various assays were performed using different pools of HUVECs and renal tubular cells. Depending on the assay, each experiment was conducted in at least duplicate using different cell pools, and each reading was repeated at least twice. Untreated cells were used as controls.

### 4.7. Cell Cultures

HUVECs were purchased from ATCC (Manassas, VA, USA) (catalog No. CRL-1730™), while primary human renal tubular cells were kindly provided by Prof. Giuseppe Cappellano. These cells were cultured in Dulbecco’s Modified Eagle’s Medium (DMEM, Sigma-Aldrich, Milan, Italy) with 10% fetal bovine serum (FBS; Euroclone, S.p.A.; Pero, Milan, Italy), 2 mM L-glutamine (Euroclone), and 1% penicillin/streptomycin (Sigma-Aldrich) [57,58,59].

### 4.8. Cell Viability

Cell viability of HUVECs and renal tubular cells was assessed using the 3-[4,5-dimethylthiazol-2-yl]-2,5-diphenyl tetrazolium bromide (MTT; Life Technologies Italia, Monza, Italy) assay [54,55,56,57,58,59,60,61]. To perform this analysis, 50,000 HUVECs/renal tubular cells/well were plated in 24-well Transwell plates in complete medium (DMEM supplemented with 10% FBS). After stimulation with plasma as described above, the medium was replaced with phenol red-free and FBS-free medium. Then, the MTT was added to each well and incubated for 2 h at 37 °C. The medium was then replaced with dimethyl sulfoxide (DMSO; Sigma-Aldrich) to dissolve the formazan crystals. The absorbance in each sample was measured at 570 nm through a spectrophotometer (VICTOR™ X Multilabel Plate Reader). The viability of HUVECs/renal tubular cells was expressed as a percentage relative to untreated control cells, which were set to 100%.

### 4.9. Mitochondrial Membrane Potential Measurement

A JC-1 assay was employed to examine the mitochondrial membrane potential of HUVECs and renal tubular cells [54,55,56,57,58,59,60,61]. Briefly, HUVECs and renal tubular cells (50,000 cells/well) seeded in 24-well Transwell plates in complete medium were stimulated with plasma for 3 h, as described above. After stimulation, the medium was removed and cells were incubated for 15 min at 37 °C with 5,51,6,61-tetrachloro-1,11,3,31 tetraethylbenzimidazolylcarbocyanine iodide (JC-1) (1X) in Assay Buffer 1X (Cayman Chemical). After two washes with assay buffer 1X, the red (excitation 550 nm/emission 600 nm) and green (excitation 485 nm/emission 535 nm) fluorescence was measured using a spectrophotometer (VICTOR™ X Multilabel Plate Reader). Data were normalized to untreated control cells.

### 4.10. MitoROS Release

The mitoROS release was measured using the Cayman’s Mitochondrial ROS Detection Assay Kit (Cayman Chemical) [54,55,56,57,58,59,60,61]. Specifically 50,000 HUVECs and renal tubular cells/well were seeded in 24-well Transwell plates in complete medium and stimulated with plasma, as described previously. After stimulation, the reaction was stopped by replacing the culture medium with 120 µL of cell-based assay buffer. Then, the buffer was aspirated, and 100 µL of Mitochondrial ROS Detection Reagent Staining Solution was added to each well. After a 20 min incubation, protected from light, the staining solution was removed, and each well was washed three times with 120 µL of PBS. Fluorescence in each sample was measured at excitation/emission wavelengths of 480 nm and 560 nm, respectively, using a spectrophotometer (VICTOR™ X Multilabel Plate Reader). Data were normalized to untreated control cells.

### 4.11. NO Release

NO release in HUVECs was quantified using the Griess assay (Promega) [54,55,56,57,58,59,60,61]. HUVECs (50,000 cells/well) were seeded in 24-well Transwell plates in complete medium and stimulated with plasma, as described previously. Finally, an equal volume of Griess reagent was added to the sample supernatants and the absorbance of each sample was measured at 570 nm, using a spectrophotometer (VICTOR™ X Multilabel Plate Reader). The NO concentration was quantified using a nitrite standard curve and expressed as μM.

### 4.12. EndMT Analysis

EndMT was assessed by fluorescence-activated cell sorting (FACS) analysis. HUVECs (50,000 cells/well) were seeded in 24-well Transwell plates in complete medium and stimulated with plasma, as described previously. After treatment, cells were detached and resuspended in 100 μL of filtered physiological solution. Subsequently, cells were incubated with 10 μg/mL of anti-CD31- phycoerythrin (PE) (Thermo Fisher Scientific, Rodano, Milan, Italy) and anti-vimentin-PE (Thermo Fisher Scientific) antibodies for 1 h at 4 °C in the dark. Data were acquired using an Attune™ NxT flow cytometer (Thermo Fisher Scientific) [45].

### 4.13. Nrf2 Expression

Renal tubular cells (50,000 cells/well) were seeded in 24-well Transwell plates containing complete medium (DMEM supplemented with 10% FBS) and stimulated with plasma as described previously. After treatment, cells were collected and resuspended in 100 µL of filtered physiological solution to achieve a final concentration of 1 × 10^6^ cells/mL. Nrf2 expression was analyzed by flow cytometry [61]. Briefly, cells were incubated with 10 µg/mL of a fluorescein isothiocyanate (FITC)-conjugated anti-Nrf2 antibody (LSBio, Seattle, WA, USA) for 1 h at 4 °C in the dark. Samples were then analyzed using an Attune™ NxT flow cytometer (Thermo Fisher Scientific).

### 4.14. Klotho Expression

Renal tubular cells (50,000 cells/well) were cultured in 24-well Transwell plates and stimulated with plasma as described previously. After incubation, cells were gently detached and resuspended in 100 µL of filtered physiological solution to reach a final density of 1 × 10^6^ cells/mL. Klotho expression was assessed by incubating cells with 10 µg/mL of an FITC-conjugated anti-Klotho antibody (Fabgennix International Inc, San Antonio, TX, USA) and incubated for 1 h at 4 °C in the dark. Data were acquired using the Attune™ NxT flow cytometer (Thermo Fisher Scientific) [62].

### 4.15. Annexin V Quantification

Annexin V levels in renal tubular cell culture supernatants were measured using the Human Annexin V ELISA Kit (Thermo Fisher Scientific) [63]. Blank wells received 100 µL of sample diluent, while sample wells received 50 µL of sample diluent and 50 µL of plasma sample, all in duplicate. A seven-point standard curve was generated by serial dilution, ranging from 100 ng/mL to 0.78 ng/mL. After loading samples and standards, 50 µL of biotin-conjugate was added to each well, and the plate was incubated at room temperature (18–25 °C) for 2 h. After incubation, wells were washed four times with 1X wash buffer, and 100 µL of diluted streptavidin–horseradish peroxidase (HRP) was added to all wells. The plate was then incubated for 1 h at room temperature, followed by another four wash cycles. Subsequently, 100 µL of 3,3′,5,5′-tetramethylbenzidine substrate solution was added to each well and incubated for 10 min in the dark. The reaction was stopped by adding 100 µL of Stop Solution, and absorbance was measured immediately at 450 nm using a VICTOR™ X Multilabel Plate Reader. Annexin V concentrations (ng/mL) were calculated based on the standard curve.

### 4.16. SIRT1 Quantification

SIRT1 levels in renal tubular cells culture supernatants were quantified using the Human SIRT1 ELISA Kit (Thermo Fisher Scientific), according to the manufacturer’s instructions. Standards were prepared by reconstituting the lyophilized standard in 400 µL of assay diluent C to obtain a stock concentration of 300 ng/mL. Serial dilutions were performed to generate a standard curve ranging from 300.0 to 1.229 ng/mL, with assay diluent C serving as the zero standard. In brief, 100 µL of each standard or diluted sample was added to the pre-coated wells. Plates were covered and incubated for 2.5 h at room temperature or overnight at 4 °C with gentle shaking. After incubation, the wells were washed four times with 1X wash buffer. Subsequently, 100 µL of 1X biotin conjugate was added to each well and incubated for 1 h at room temperature with shaking, followed by another four wash cycles. Next, 100 µL of streptavidin–HRP solution was added to all wells and incubated for 45 min at room temperature. After a final wash step, 100 µL of TMB substrate solution was added and incubated for 30 min in the dark. The reaction was stopped by adding 50 µL of stop solution, and absorbance was measured immediately at 450 nm using a VICTOR™ X Multilabel Plate Reader. SIRT1 concentrations (ng/mL) were calculated from the standard curve using a four-parameter logistic regression model.

### 4.17. Statistical Analysis

For each patient, the mean of the multiple measurements was considered for the analysis. All data are presented as the medians and range (maximum–minimum) of different experiments. Differences between groups were assessed using the Mann–Whitney test, while differences between time points were assessed using the Wilcoxon signed-rank test. A value of *p* < 0.05 was considered statistically significant. Statistical analyses and graphs were executed by using GraphPad Prism version 9.0.0 (GraphPad Software; San Diego, CA, USA) and STATA v.19 (StataCorp. 2025 Statistical Software: Release 19; College Station, TX, USA).

## 5. Conclusions

CSA-AKI remains a major clinical challenge, profoundly impacting patient outcomes and healthcare costs. The findings of the present study support a complex pathophysiologic framework in which pre-existing oxidative stress, inflammation, and endothelial dysfunction may contribute to patient susceptibility, while cardiac surgery may further amplify these mechanisms in those who eventually develop AKI. In this context, our data support the presence of circulating injurious factors in patient plasma even before surgery, with a further worsening after surgery only in the AKI group. These findings indicate associations between circulating factors and cellular dysfunction, although causality cannot be established from this in vitro model, which cannot reflect the full neurohumoral and hemodynamic complexity of the in vivo perioperative setting.

These observations are clinically relevant because currently used biomarkers, such as serum creatinine and urine output, are poorly sensitive to early and subclinical injury. Still-unidentified circulating factors may therefore represent additional tools for the early identification of patients at risk and may support more timely preventive strategies.

Overall, our results expand current knowledge of CSA-AKI pathophysiology and may support the development of personalized care protocols, as well as the optimization of surgical equipment, including pulsatile perfusion systems, microemboli filters, and biocompatible circuits, with the aim of reducing systemic inflammatory and oxidative responses and limiting the release of still-unidentified circulating factors. A better understanding of the mediators involved may also help guide future strategies to reduce inflammatory and oxidative injury during cardiac surgery, including further optimization of extracorporeal circulation and related surgical technologies.

## Data Availability

Database access will be restricted to the coordinating team. External researchers may obtain access only with prior approval from the principal investigator, based on review of their research proposal and analysis plan.

## Supplementary Materials

The following supporting information can be downloaded at https://www.mdpi.com/article/10.3390/ijms27104416/s1.
