# Supplementary figures and images for: Plasma from Cardiac Surgery Patients Induces Endothelial and Tubular Epithelial Cell Damage: Potential Role in Acute Kidney Injury Development—A Preliminary Report
Source: Int J Mol Sci. 2026 May 15;27(10):4416. doi: 10.3390/ijms27104416 (PMC13207256; doi:10.3390/ijms27104416)

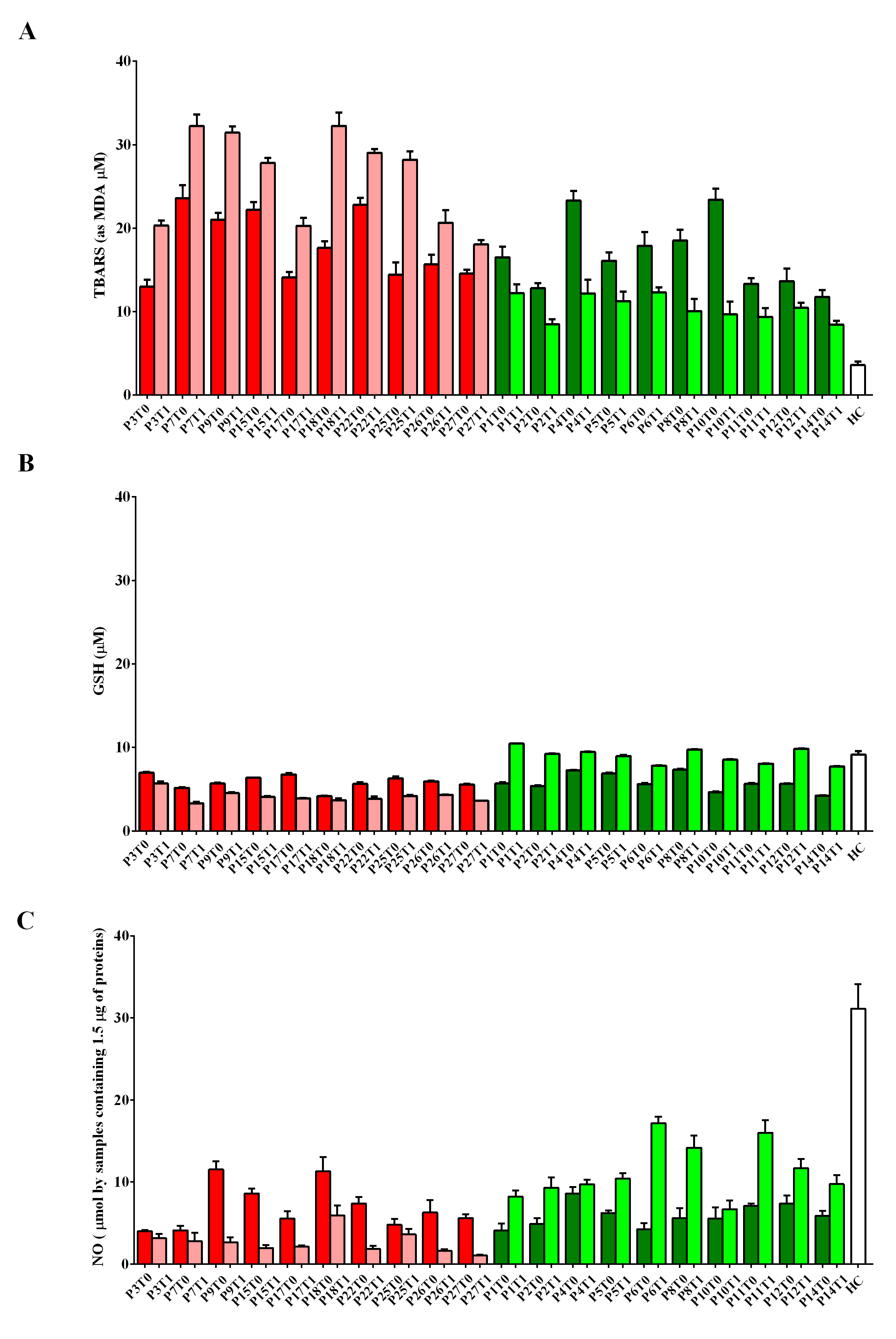

Supplement: Supplementary file 1 [file ijms-27-04416-s001.zip › ijms-4252476-supplementary/Supplemental/Fig S1.jpg]

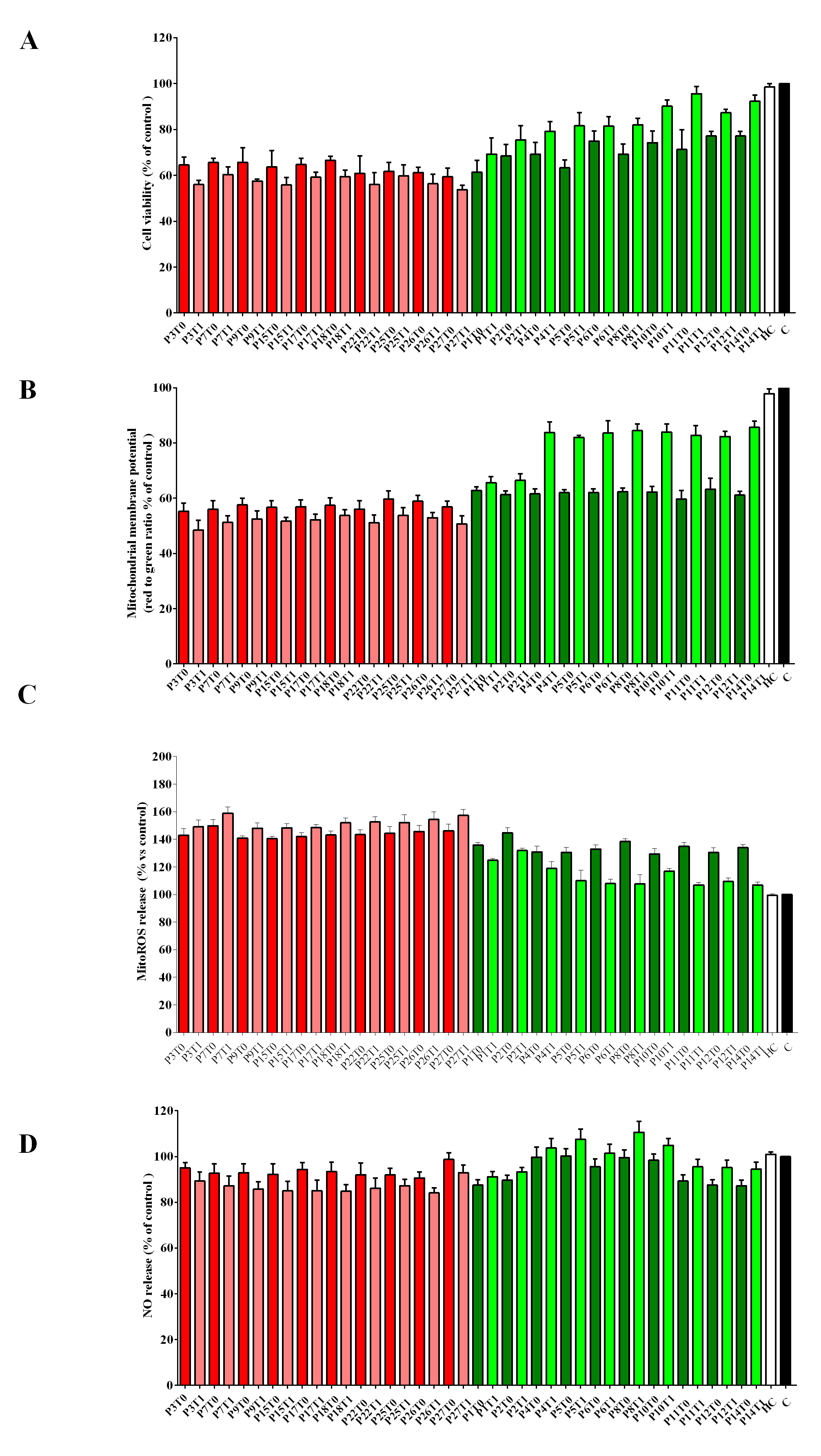

Supplement: Supplementary file 1 [file ijms-27-04416-s001.zip › ijms-4252476-supplementary/Supplemental/Fig S2.jpg]

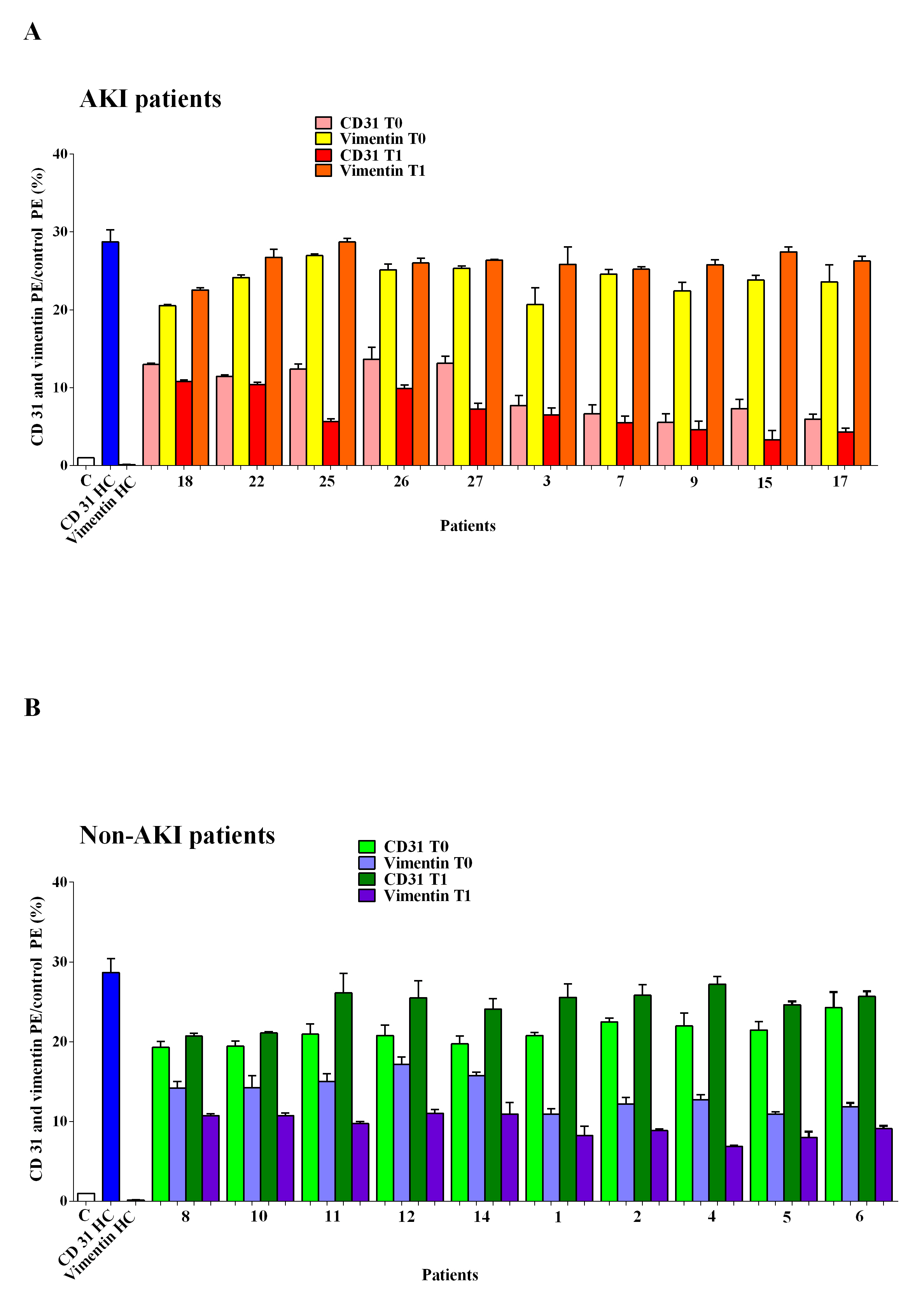

Supplement: Supplementary file 1 [file ijms-27-04416-s001.zip › ijms-4252476-supplementary/Supplemental/Fig S3.jpg]

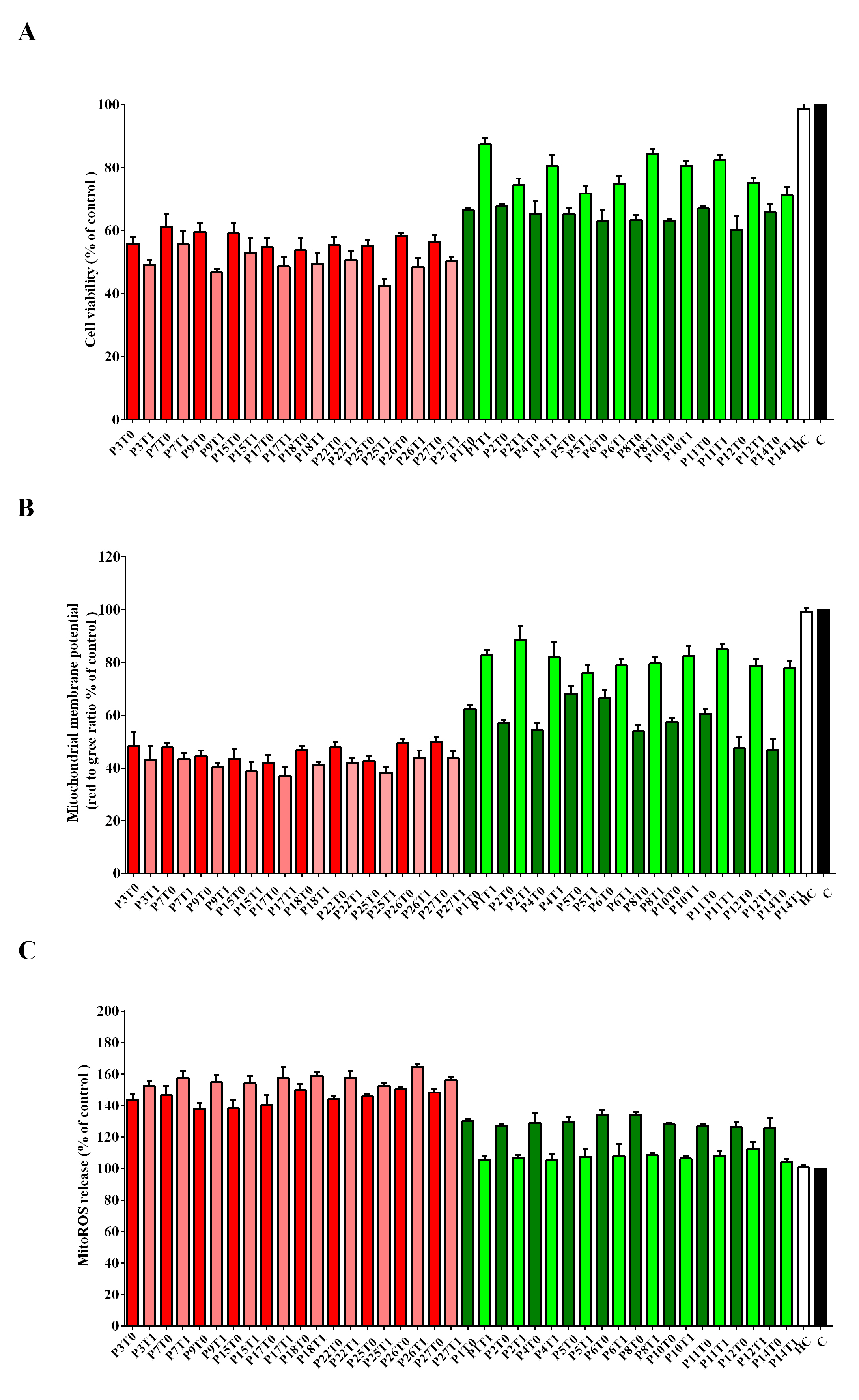

Supplement: Supplementary file 1 [file ijms-27-04416-s001.zip › ijms-4252476-supplementary/Supplemental/Fig S4.jpg]

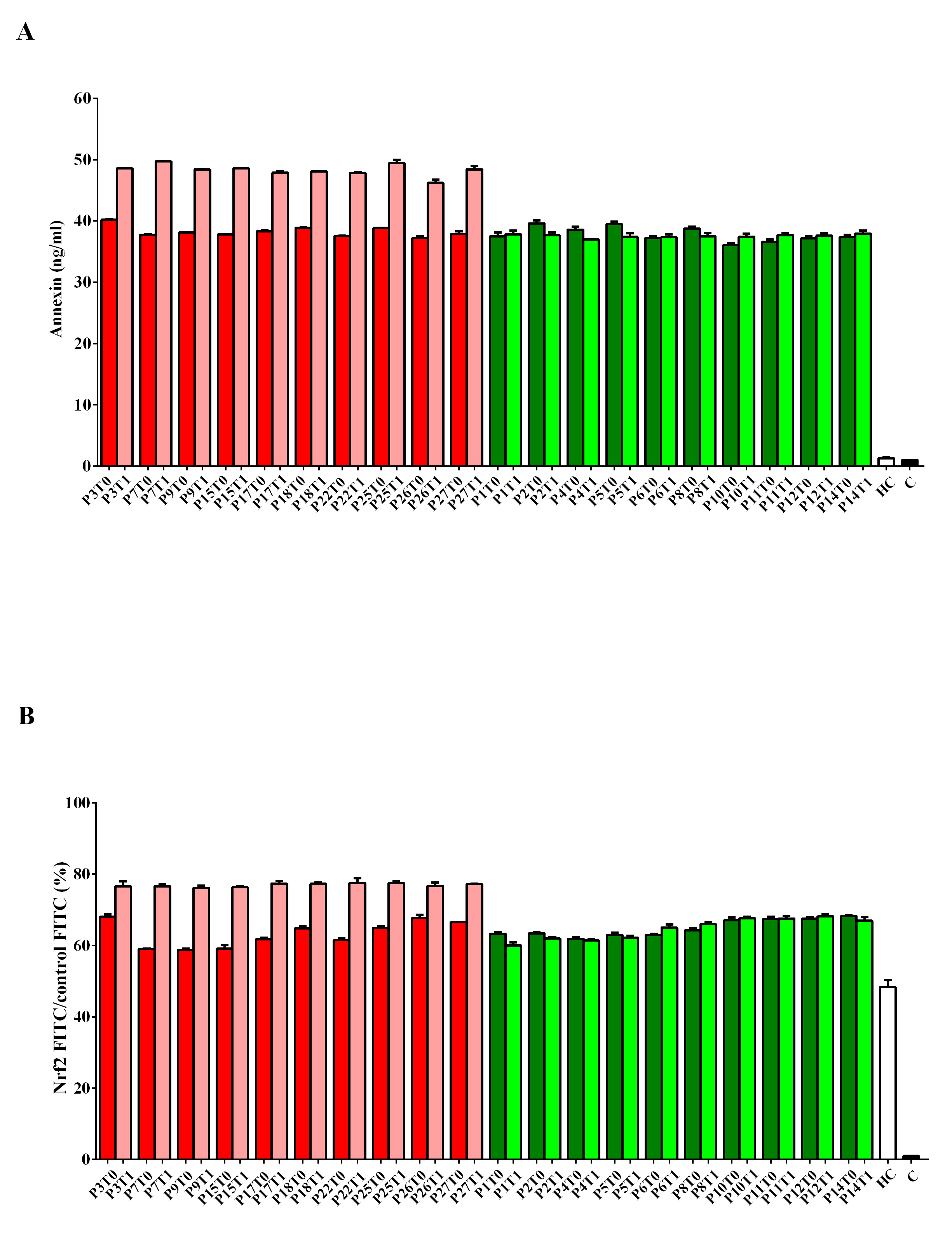

Supplement: Supplementary file 1 [file ijms-27-04416-s001.zip › ijms-4252476-supplementary/Supplemental/Fig S5.jpg]

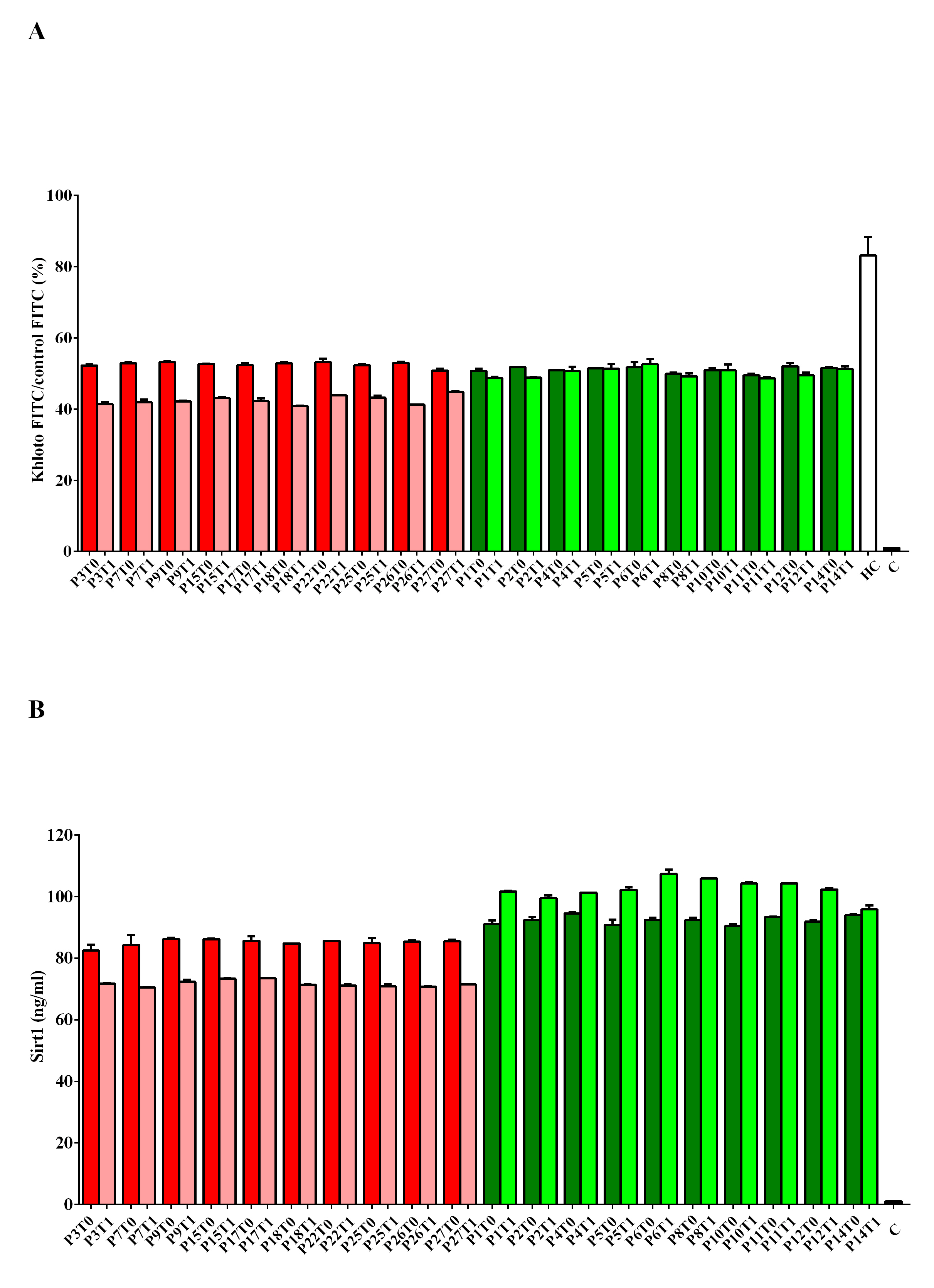

Supplement: Supplementary file 1 [file ijms-27-04416-s001.zip › ijms-4252476-supplementary/Supplemental/Fig S6.jpg]
